# Juvenile onset autoinflammatory disease due to a novel mutation in *TNFAIP3 (A20)*

**DOI:** 10.1186/s13075-018-1766-x

**Published:** 2018-12-10

**Authors:** Shuzo Sato, Yuya Fujita, Tomonari Shigemura, Hisanori Matoba, Kazunaga Agematsu, Yuya Sumichika, Makiko Yashiro, Atsushi Ono, Yukihiko Kawasaki, Hiroko Kobayashi, Hiroshi Watanabe, Tomohiro Koga, Atsushi Kawakami, Kiyoshi Migita

**Affiliations:** 1 Department of Rheumatology, Fukushima Medical University School of Medicine, 1 Hikarigaoka, Fukushima, Fukushima 960-1295 Japan; 2 Department of Infectious Immunology, Shinshu University Graduate School of Medicine, Asahi 3-1-1, Matsumoto, Nagano, 390-8621 Japan; 3 Department of Pediatrics, Fukushima Medical University School of Medicine, 1 Hikarigaoka, Fukushima, Fukushima 960-1295 Japan; 4 Department of Immunology and Rheumatology, Unit of Translational Medicine, Graduate School of Biomedical Sciences, Nagasaki University, Sakamoto1-7-1, Nagasaki, 852-8501 Japan

## Key message

Novel heterozygous C200A *A20/TNFAIP*3 gene mutation is responsible for autosomal-dominant juvenile onset autoinflammatory disorder.

## Letter

A20, which is encoded by the *TNFAIP3* gene, has been shown to control nuclear factor kappa B (NF-κB) signalling by deubiquitinating receptor-interacting proteins [1]. Recently, heterozygous mutations in the *TNFAIP3* gene have been found to cause the haploinsufficiency of A20, which presents as an early-onset autoinflammatory disease [2, 3]. Here, we report a Japanese family containing two cases of autoinflammatory disease which exhibit an identical novel *TNFAIP3* mutation.

The patient was a 17-year-old Japanese boy who was referred to our department with a 3-year history of recurrent painful oral ulcer and epigastralgia, accompanied by low-grade fever. He presented with periodic fever and oral aphtha that had occurred for the prior 3 years and was referred to our hospital. After spontaneous resolution of the symptoms, he was regularly followed in our hospital. Three years later, the patient was admitted to our hospital for impaired food intake due to epigastralgia, oral ulcer, and fever. His mother had exhibited oral and genital ulcers and erythema nodosum-like lesions on the upper extremities and had died of septicaemia 2 years prior. His younger sister exhibited similar symptoms from the age of 11 years and was suspected of Behçet’s disease. Physical examination of the boy revealed painful oral ulcers. Neither genital ulcer nor uveitis was confirmed, but multiple erosions in his stomach mucosa were observed by endoscopic examination.

Relevant laboratory data are listed in Additional file 1 (Table S1). There were no abnormalities except elevated levels of C-reactive protein (CRP). Anti-nuclear antibodies were positive with low titres; however, tests for other various autoantibodies revealed no abnormalities. The HLA-B haplotype of the patient was not B51 (B39/B62). Increased serum inflammatory cytokine levels were confirmed at the time of presentation (Additional file 1: Table S1). Although autoimmune phenomena were not prominent, this patient presented with an autoinflammatory phenotype resembling Behçet’s disease. Treatment with prednisolone relieved the clinical symptoms and normalized CRP levels.

Recently, Zhou et al. reported 11 patients from six families with a new dominantly inherited autoinflammatory disease, termed haploinsufficiency of A20 (HA20) [4]. This disease has the following characteristics: periodic fever; oral, ocular, and/or genital ulcers; and elevated levels of many cytokines [4]. Serum pro-inflammatory cytokines, particularly tumour necrosis factor (TNF)-α, interleukin (IL)-1β, IL-6, and IL-8, were elevated in our patient (Additional file 1: Table S1). Therefore, written informed consent for *TNFAIP3* gene analysis was obtained from the patient and his father.

**Fig. 1:**
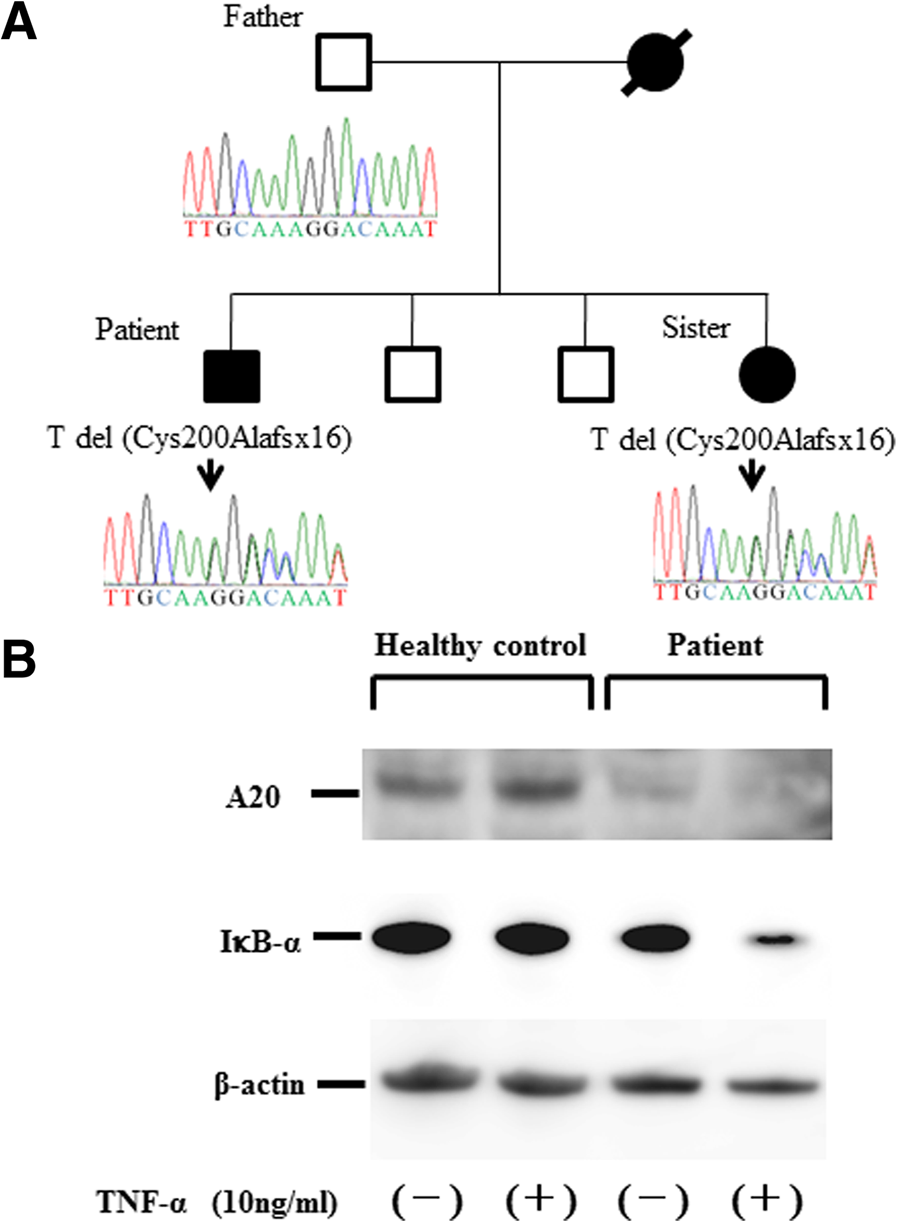
**a** Family tree and *A20/TNFAIP3* mutations. Sequence analysis of the A20/TNFAIP3 gene among the proband and family members showed a heterozygous mutation of C200A in exon 4, which was absent in the healthy father. The c597-598 T deletion variant resulted in a frameshift and premature stop codon (C200A fs*16) in the OTU domain of A20. Sequence analysis was not possible on the younger brothers of the proband without symptoms. Sequence analysis results are shown using reverse primer. **b** C200A fs*16 mutation reduced A20 in peripheral blood mononuclear cells (PBMCs). PBMCs isolated from the proband (patient) and a healthy control were stimulated with tumour necrosis factor alpha (TNF-α; 10 ng/ml) for 24 h and whole cell lysates were immunoblotted for representative target proteins. Reduced A20 protein expressions in proband PBMCs were confirmed at baseline and after TNF-α-stimulation (24 h). Cytosolic expression levels of inhibitor of nuclear factor kappa B alpha (IκB-α) were reduced by TNF-α stimulation in the patient’s PBMCs compared with those in control PBMCs

The C596_598 del A mutation, which we confirmed by Sanger sequencing (Fig. 1a), introduces a frameshift substitution of alanine for cysteine at position 200, generating a downstream stop codon (Cys200Alafsx16; C200Afs*16) in the OTU domain of A20. This heterozygous C200A fs*16 mutation was present in the patient’s younger sister, who showed similar symptoms. Sanger sequencing of the unaffected father showed no variant of *TNFAIP3*, suggesting that these mutations were maternally derived. This variant is absent from public databases (ExAs/dbSNP).

To address the molecular basis of this C200Afs*16 mutation, we stimulated peripheral blood mononuclear cells (PBMCs) that were isolated from the patient and from a healthy subject with TNF-α (10 ng/ml), and then performed immunoblot blot analysis with anti-A20 antibodies (Fig. 1b). Wild-type A20 protein expression was reduced in patient-derived PBMCs compared with those from the healthy subject at baseline and after TNF-α stimulation. These results suggested that the frameshift mutation identified in the present case resulted in impaired expression of A20 at baseline and after TNF-α stimulation. Additionally, cytosolic inhibitor of nuclear factor kappa B alpha (IκB-α) protein levels were reduced in patient-derived PBMCs after stimulation with TNF-α, whereas IκB-α protein levels were unchanged in control PBMCs. These findings suggest that IκB-α degradation was accelerated by TNF-α stimulation in PBMCs isolated from the patient.

A20 is a ubiquitin-editing enzyme that inhibits key proinflammatory molecules, including inhibitor of nuclear factor kappa B kinase (IKK)γ [5]. Zhou et al. reported that unrelated family cases manifested autoinflammation with heterozygous mutations in *TNFAIP3*; all patients exhibited oral and genital ulcers [4]. The clinical manifestations seen in our patients were consistent with those of the previously reported cases [6], whereas the disease onset (at 14 years old) was relatively late compared with those of the reported cases [6]. We identified a novel heterozygous missense mutation, which was located at the OTU domain of A20 in both affected siblings in this family.

In previously reported HA20 cases, mutant cells have shown enhanced NF-κB activity, as demonstrated by increased phosphorylation of IKKα/β and increased degradation of IκB-α [7]. In the PBMCs isolated from our patient, cellular IκB-α failed to maintain baseline levels compared with cells expressing wild-type A20 in response to extended TNF-α stimulation. IKKα/β mediated phosphorylation of IκB-α resulted in IκB-α degradation and subsequent nuclear translocation of NF-κB [8]. These findings suggest that haploinsufficiency of *TNFAIP3*, which leads to impaired A20 expression, could be responsible for the TNF-α-induced increased degradation of IκB-α in the present case. Although the molecular pathogenesis of our patient and previously reported cases may be similar, variations in clinical manifestations suggest additional modifying factors [6]. Further studies regarding functional and genetic analysis of A20 will elucidate the pathogenesis of A20-mediated autoimmune/autoinflammatory diseases.

## Additional file

Additional file 1: **Table S1.** Laboratory findings and cytokine/chemokine profile on admission. (PDF 131 kb)

## Abbreviations

HA20: Haploinsufficiency of A20
IKK: Inhibitor of nuclear factor kappa B kinase
IL: Interleukin
IκB-α: Inhibitor of nuclear factor kappa B alpha
PBMC: Peripheral blood mononuclear cell
TNF: Tumour necrosis factor
