# Supplementary material for: Juvenile onset autoinflammatory disease due to a novel mutation in TNFAIP3 (A20)
Source: Arthritis Res Ther. 2018 Dec 10;20:274. doi: 10.1186/s13075-018-1766-x (PMC6288856; doi:10.1186/s13075-018-1766-x)
Supplement: Supplementary file 1 — Table S1. Laboratory findings and cytokine/chemokine profile on admission. (PDF 131 kb) [file 13075_2018_1766_MOESM1_ESM.pdf]

Laboratory Findings and Cytokine/Chemokine profile on Admission

**Peripheral blood**		**Serological tests**		**Cytokine/Chemokine profile**		Healthy(n=40) median(interquartile range)
Red blood cells	427×10^4 /μL	C-reactive protein	0.55 mg/dL (<0.30)	IL-1β	6.46 pg/mL	0.9(0.3-1.5) pg/mL
Hemoglobin	12.9 g/dL	Erythrocyte sedimentation rate	8 mm/hr (<15)	IL-1RA	44.66 pg/mL	38.7(27.5-68.7) pg/mL
Hematocrit	39.3%	sIL-2R	315 U/ml (121-613)	IL-2	5.57 pg/mL	1.1(0.5-2.1) pg/mL
Plt	17.9×10^4 /μL	Serum amyroid A	30 μg/dL (<8.0)	IL-4	326.56 pg/mL	1.0(0.11-4.2) pg/mL
White blood cells	7,700 /μL	IgG	1,583 mg/dL (870-1700)	IL-5	2.48 pg/mL	1.1(0.9-1.5) pg/mL
Neutriphil	51.0%	IgA	355 mg/dL (110-410)	IL-6	27.85 pg/mL	0.6(0.1-1.9) pg/mL
Eosinophil	2.0%	IgM	92 mg/dL (35-220)	IL-7	20.21 pg/mL	6.0(2.4-9.6) pg/mL
Monocyte	11.0%	C3	102 mg/dL (65-135)	IL-8	158.63 pg/mL	36.0(14.0-112.4) pg/mL
Lymphocyte	35.0%	C4	25 mg/dL (13-35)	IL-10	14.73 pg/mL	1.4(0.1-2.5) pg/mL
Baso	1.0%	ANA	160 Homogeneous (<×40)	IL-12p40	84.44 pg/mL	8.9(0.9-16.7) pg/mL
**Blood chemistry**		Anti-ds-DNA Ab	(-) (<9.9)	IL-12p70	8.10 pg/mL	4.5(3.0-9.5) pg/mL
Total protein	7.3 g/dL	Anti-sm Ab	(-) (<6.9)	IL-17	5.69 pg/mL	4.7(2.5-7.8) pg/mL
Total bilirubin	0.7 mg/dL	Anti-U1RNP Ab	(-) (<4.9)	TNF-α	211.66 pg/mL	7.2(5.3-.8.9) pg/mL
Albumin	4.3 g/dL	Anti-SSA Ab	(-) (<6.9)	IFN-γ	35.33 pg/mL	13.08(6.9-22.4) pg/mL
Glutamic-oxaloacetic transaminase	13 IU/L (13-33)	Anti-SSB Ab	(-) (<6.9)	G-CSF	102.16 pg/mL	24.3(21.5-36.5) pg/mL
Glutamic-pyruvic transaminase	5 IU/L (8-42)	PR3-ANCA	(-) (<2.0 U/mL)	GM-CSF	31.08 pg/mL	17.7(13.3-31.5) pg/mL
Lactate dehydrogenase	127 IU/L (260-119)	MPO-ANCA	(-) (<3.5 U/mL)	VEGF	356.35 pg/mL	172.72(111.4-270) pg/mL
Alkaline phosphatase	424 IU/L (80-250)	HBs Ag	(-)	FGF-2	115.48 pg/mL	70.5(55.5-88.8 pg/mL
Creatine Kinase	51 IU/L (62-287)	HCV Ab	(-)	CCL2	898.06 pg/mL	596.31(482.0-789.5) pg/mL
Blood urea nitrogen	9 mg/dL	**Urinalysis**	normal	CCL3	238.99 pg/mL	10.6(5.7-14.7) pg/mL
Cr	0.69 mg/dL			CCL4	384.69 pg/mL	65.3(38.9-87.0) pg/mL
Na	141 mEq/L	**HLA type**	A2, A51	CCL22	2,238.55 pg/mL	860.6(633.0-968.9) pg/mL
K	4.0 mEq/L		B62, B39	CXCL10	1,364.74 pg/mL	218.4(178.6-302.2) pg/mL
Cl	106 mEq/L		DR4, DR14	CX3CL1	181.12 pg/mL	92.0(67.3-145.8) pg/mL

Abbreviation: HBsAg; hepatitis B virus surface antigen, HCV; hepatitis C virus, MPO-ANCA; myeloperoxidase-antineutrophil cytoplasmic antibody, PR3-ANCA; proteinase 3-antineutrophil cytoplasmic antibody, sIL-2R; soluble interleukin-2 receptor, IL; interleukin, RA; recepter antagonist, CCL; chemokine ligand, TNF; tumor necrosis factor, IFN; interfeon, G-CSF; granulocyte colony stimulating factor, GM-CSF; granulocyte macrophage colony stimulating factor, EGF; epidermal growth factor, VEGF; vascular endotherial growth factor, FGF; fibroblast growth factor.
